# Sex-dependent responses to moderate/low-intensity swimming and loaded ladder-climbing resistance exercise in mdx mice

**DOI:** 10.1007/s10974-026-09734-1

**Published:** 2026-06-24

**Authors:** Luana Lima Rocha da Silva, Tabata Gabriely Pereira Barbosa de Souza, Joice Luiza Silva Cassini, Beatriz Godinho Nascimento, Luis Felipe Cunha dos Reis, Thaís de Sousa Máximo, Túlio de Almeida Hermes

**Affiliations:** 1 Departament of Anatomy, Federal University of Alfenas, – Unifal-MG, 700 Gabriel Monteiro da Silva, St. Alfenas – Minas Gerais, 37130-001 Alfenas, Brazil; 2 Department of Structural Biology, Federal University of Alfenas (UNIFAL- MG), Alfenas, MG Brazil

**Keywords:** Duchenne muscular dystrophy, mdx mouse, Exercise, Sex differences, Swimming, Resistance training

## Abstract

Duchenne muscular dystrophy (DMD) is a severe genetic disorder characterized by progressive muscle degeneration, and the mdx mouse has been widely used to investigate disease mechanisms and therapeutic strategies, including the impact of different exercise modalities and sex-related responses on dystrophic muscle. This study compared moderate/low-intensity swimming and loaded ladder-climbing in male and female mdx, including sedentary control groups for each sex. Animals were subjected to a 4-week swimming (30 min/day, 4 days/week) or a 15-day ladder-climbing resistance protocol (6 sessions, 3 sets of 10 climbs, every 48 h), starting at 30 and 45 days of age, respectively. All groups (male and female sedentary controls, swimming, and resistance-trained mice) were evaluated at 60 days of age. In male mdx mice, swimming reduced serum creatine kinase levels and improved limb functional performance compared to sedentary controls. In contrast, resistance exercise increased diaphragmatic fibrosis and reduced centrally nucleated fibers in biceps brachii in male mdx mice. Resistance-trained males also exhibited lower holding impulse compared to swimming-trained males. Females exhibited a milder response, with superior fatigue resistance, greater mechanical work during climbing, and superior final holding impulse compared to males. These findings demonstrate that exercise outcomes in mdx mice depend strongly on modality, intensity, and sex. Swimming provided functional benefits, while ladder-climbing induced measurable but controlled muscle adaptations, without consistent evidence of widespread damage.

## Introduction

The mdx mouse (C57BL/10 mdx/*X chromosome-linked muscular dystrophy*), a widely used experimental model of Duchenne muscular dystrophy (DMD), carries a nonsense mutation in exon 23 of the dystrophin gene, resulting in the absence of dystrophin and leading to continuous cycles of myofiber degeneration and regeneration (Bulfield et al. 1984; Yucel et al. 2018). They present a marked inflammatory infiltrate in areas of myonecrosis but differ from the human phenotype in that they do not develop intense fibrosis or adipose tissue accumulation (Cullen and Jaros 1988; Yucel et al. 2018).

Forced exercise in 6-12-week-old mdx mice is commonly employed to exacerbate muscle necrosis and elevate creatine kinase (CK) levels, more accurately recapitulating the severe phenotype of human DMD (Grounds et al. 2008; McGeachie et al. 1993). For instance, higher-intensity protocols (≥ 12 m/min or involving uphill/downhill running) promoted extensive collagen deposition and fibrosis, upregulated key mediators of the degeneration–regeneration cycle in mdx skeletal muscle (phosphorylated ERK1/2, p38 MAPK, and JNK2), and exacerbated sarcolemmal permeability and calcium influx, leading to increased oxidative stress, inflammation, and extensive myofiber necrosis (Frinchi et al. 2021). In contrast, low-intensity training is beneficial. The expression of mitochondrial differentiation genes (Baltgalvis et al. 2012; Hulmi et al. 2013) and muscle mass increased after voluntary wheel running in animals aged 4 to 8 weeks. Swimming and low-intensity running in young mdx mice also stimulated a shift from fast glycolytic (type IIb) muscle to oxidative (type IIa) and slow (type I) muscle (Landisch et al. 2008; Matsakas et al. 2013). Swimming, with a variable duration of up to 10 weeks, appears to produce adaptations to functional demand and beneficial effects on the limb muscles of mdx mice, regardless of the intensity of the exercise (Frinchi et al. 2021).

Sex is another significant factor influencing responses to physical exercise in mdx mice. Males typically display greater strength and faster contractile properties, whereas females show higher fatigue resistance, reduced susceptibility to exercise-induced damage, and often exhibit a milder phenotype with decreased muscle damage, indicating sex-related differences in disease progression and muscle pathology (Glenmark et al. 2004; Grounds et al. 2008). These differences are mainly attributed to estrogen, which has been shown to promote muscle repair in both healthy and mdx mice (Grounds et al. 2008; Tiidus 2001; Tiidus et al. 2001). In a previous study, we found that intense treadmill exercise increased the expression of estrogen α and β receptors in the diaphragm (DIA) muscle of mdx mice, especially in females (Hermes et al. 2018). This was associated with less muscle damage in females compared to males, suggesting that exercise may enhance estrogen-mediated protective mechanisms in dystrophic muscle. Therefore, because males and females show different adaptations to high-intensity running, it is essential to assess how other exercise modalities, such as moderate/low-intensity swimming and resistance training, affect both sexes.

Considering that physical training in mdx mice can be used to evaluate their functional capacity, investigate the effects of exercise on dystrophic muscle, or exacerbate the phenotype prior to therapeutic interventions (Hyzewicz et al. 2015a), the present study aims to compare the effects of medium- to low-intensity exercise (swimming) and resistance exercise (loaded ladder-climbing) on functional and morphological parameters in male and female mdx mice.

## Materials and methods

### Animals

**Fig. 1:**
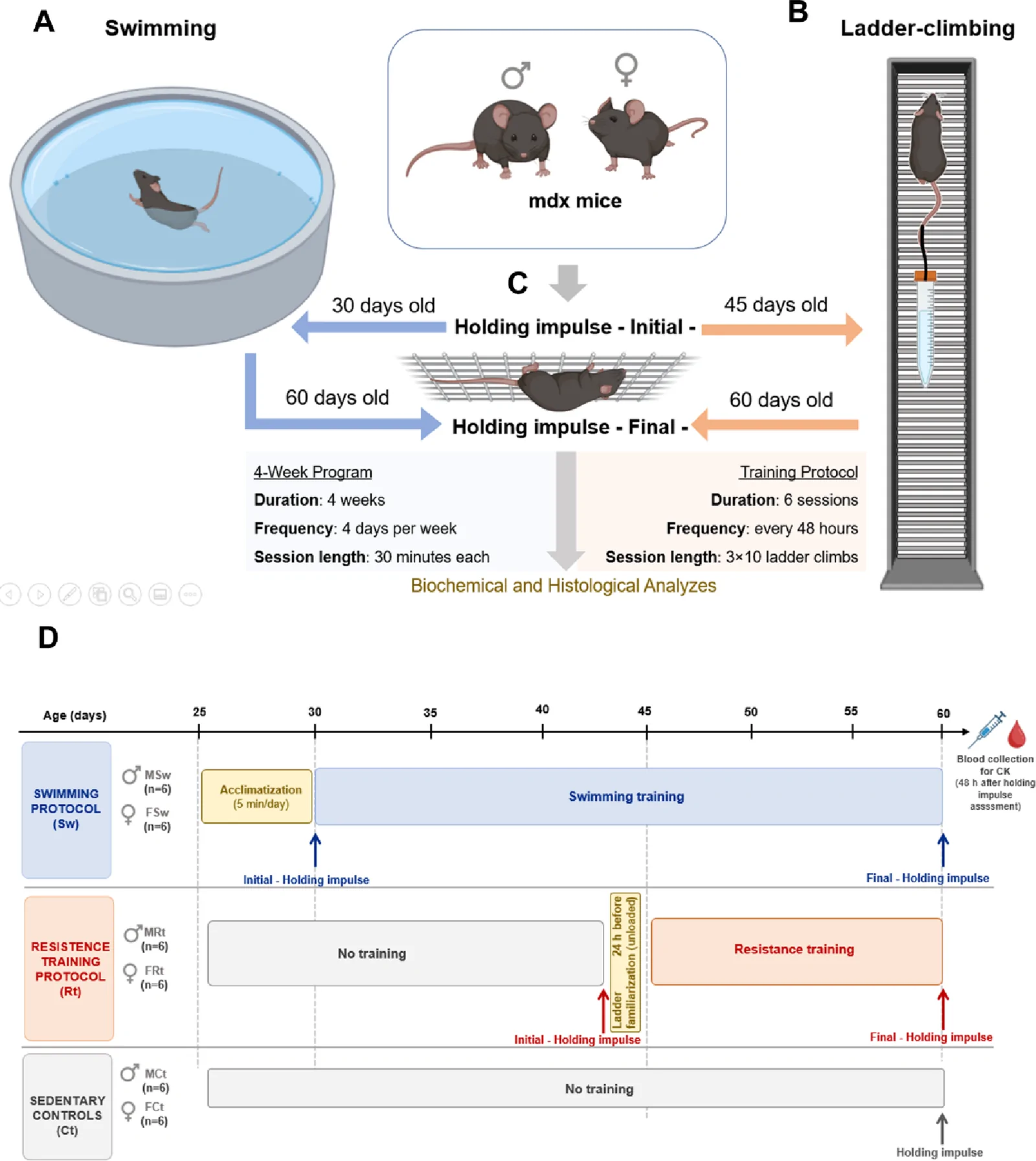
Experimental design and timeline for the physical training protocols in mice. The study employed two distinct training modalities over a developmental period from 30 to 60 days of age. (**A**) Swimming Protocol: Mice began the protocol at 30 days old. The training consisted of a 4-week program with sessions conducted 4 days per week, each lasting 30 min. The final assessment was performed at 60 days of age. (**B**) Resistance Training Protocol (ladder-climbing): Mice began this protocol at 45 days old. The resistance training regimen lasted 15 days and consisted of 6 training sessions administered every 48 h. Each session comprised 3 sets of 10 ladder climbs. Initial and final performance assessments, based on holding impulse, were conducted at 45 and 60 days of age, respectively. (**C**) Performance Assessment - Holding Impulse: The holding impulse (measured in g sec) was used as a key functional metric. (**D**) Experimental design and timeline for the physical training protocols in mdx mice. The updated scheme includes acclimatization/familiarization periods, exercise-training periods correlated with animal age, sedentary control groups, time points for muscle function testing (holding impulse), and blood collection for CK analysis

Mdx (C57BL/10-Dmdmdx/PasUnib) mice of both sexes, aged 30, 45, and 60 days postnatal, were used (Fig. 1). The animals were maintained in the vivarium of the Department of Anatomy at the Federal University of Alfenas (UNIFAL-MG), and the breeding pairs originated from the Multidisciplinary Center for Biological Research (CEMIB), UNICAMP. After birth, pups remained with the dam until weaning, when the male was separated. Throughout the experiment, the animals were kept in plastic cages with a 12-hour light/dark cycle, fed Nuvilab CR-1 feed, and with water ad libitum. The experimental protocols were approved by the Ethics Committee on the Use of Animals (CEUA/UNIFAL-MG; 0023/2024) and are in accordance with the ethical principles in animal experimentation adopted by the Brazilian College of Animal Experimentation (COBEA).

The animals were divided into the following experimental groups: (1) Sedentary Male (MCt): 5 male mdx mice, 60 days old, that were not subjected to exercise protocols; (2) Sedentary Female (FCt): 5 female mdx mice, 60 days old, that were not subjected to exercise protocols; (3) Male Swimming (MSw): 5 male mdx mice, 30 days old, subjected to the swimming protocol; (4) Female Swimming (FSw): 5 female mdx mice, 30 days old, subjected to the swimming protocol; (5) Male Resistance (MRt): 5 male mdx mice, 30 days old, subjected to the resistance training protocol; (6) Female Resistance (FRt): 5 female mdx mice, 30 days old, subjected to the resistance training protocol.

### Swimming protocol

Mice were introduced into a glass tank (40 × 25 × 20 cm) filled with water (maintained at 35 °C ± 1 °C) at a depth sufficient to force them to swim (Fig. 1). A researcher monitored the animals to prevent them from drowning. The animals underwent a 4-week program consisting of 30-minute exercise sessions, 4 days a week (Monday, Tuesday, Thursday, and Friday), with the remaining 3 days designated for rest (Hyzewicz et al. 2015b, 2017). The swimming protocol was also maintained with a constant duration and frequency throughout the experimental period.

Prior to the experimental protocol, mice were acclimatized to the aquatic environment through daily 5-minute swimming sessions for one week before the beginning of the training protocol, to minimize stress and facilitate adaptation.

### Resistance training protocol

The ladder-climbing protocol was adapted from Ferraresi et al. (2015), which originally described its use in wild-type/healthy mice. In the present study, the protocol was adjusted to accommodate the functional limitations of mdx mice. An inclined ladder (80°, 100 cm long, 9 cm wide; Fig. 5A) containing metal bars spaced every 0.5 cm. The maximum climbing distance was limited to 70 cm in order to avoid contact of the load with the ground during the exercise (Fig. 1).

The animals in the resistance groups (MRt and FRt) were previously familiarized with the equipment one day before the start of the muscle performance assessment and training. The familiarization procedure consisted of 2 sets of 5 climbs. Consecutive sets were performed on the ladder, with two-minute rest intervals between sets. No load was attached to the animals’ tails during this stage, aiming only at motor and behavioral adaptation to the experimental apparatus.

Twenty-four hours after familiarization, a test was conducted to determine the ideal load to be used throughout the training. Initially, the animals’ response to the weight of an empty Falcon tube (15 ml; Fig. 5A) attached to the tail with adhesive tape was evaluated, and subsequently, the tube was filled with increasing volumes of water (0.5 ml). This procedure allowed us to verify the animals’ ability to sustain and climb with different loads. We determined that the load corresponding to ~ 43% and 45% (~ 2.5 ml of water = 8.74 g) of the average body weight (for MRt and FRt, respectively) provided the ideal effort level, allowing the mice to climb with adequate resistance and movement continuity in 3 repetitions without failure (adapted from Ferraresi et al. 2015).

After 24 h from the initial load assessment, the animals underwent 6 training sessions conducted on alternate days (every 48 h). Each training session consisted of 3 sets of 10 repetitions (step-ups). The mouse was trained to climb the ladder with a 2-minute rest period between each set. During some repetitions, light pressure was applied to the mouse’s tail with tweezers to encourage the animal to climb and complete the exercise. If, after three applications of gentle pressure, the mouse was unable to resume climbing and remained stationary, the series of repetitions was interrupted. The training protocol was maintained with a consistent workload throughout the experimental period.

Throughout all stages of the experiment, the mice were observed for signs of excessive fatigue or discomfort, and the protocol was immediately interrupted in cases of repeated loss of grip, inability to continue movement, persistent panting, or any indication of distress. All procedures were conducted in accordance with ethical standards for animal experimentation, and any signs of pain or injury were promptly assessed and treated according to the institution’s ethics committee protocols.

During the ladder-climbing protocol, animals were initially assigned to perform three sets of ten climbs with the corresponding load attached to the tail. However, the actual number of repetitions completed by each animal was recorded, as some mice were unable to complete all repetitions within a set. For each session, the number of repetitions performed in each set and the time required to complete the exercise were measured. These variables were then used to calculate muscle work and muscle power for each training session.

### Muscle function test

The muscle function test was performed using the four-limb suspension test, to evaluate the effect of the exercise modalities 24 h before the first day and 24 h after the last day of protocol application (Fig. 1). The sedentary groups (MCt and FCt) received the protocol at 60 days of age.

The test was conducted in accordance with the TREAT-NMD protocol, entitled “The use of four-limb suspension testing to monitor muscle strength and condition over time” (Carlson et al. 2010; Carlson 2011). Briefly, the animals were placed individually in the center of a wire grid, which was then inverted and raised 30 cm above a box containing wood shavings. The time each animal managed to remain on the grid without falling was recorded.

*Holding Impulse = body weight (g) x time the animal held the hold (sec).*

### Functional performance

It was evaluated in ladder-climbing resistance training only, as described by Ferraresi et al. (2015). During the protocol, the number of repetitions per set, climb time, and climbing distance in incomplete attempts were recorded. These values were then used to calculate muscle work and power. Calculations considered the external load carried by the animals (8.74 g = 0.00874 kg), the vertical displacement during climbing, and the execution time of session.

The vertical displacement corresponded to the vertical component of the 70 cm climb performed on the 80° inclined ladder and was calculated as:

$${\mathrm{d}}\, = \,0.{\mathrm{7}}0{\text{ }} \times {\text{ sin}}\left( {{\mathrm{8}}0^\circ } \right).$$

resulting in a vertical displacement of 0.689 m per ascent.

Mechanical work (*W*) was calculated considering the external load only (8.74 g = 0.00874 kg), according to:

$${\mathrm{W}}\, = \,{\text{m }} \times {\text{ g }} \times {\text{ d}}.$$

where *m* is the external load (kg), *g* is the acceleration of gravity (9.81 m·s^− 2^), and *d* is the vertical displacement (m).

Thus, the average work done per ascent was approximately 0.059 J. Total work (*totalW*) per session was calculated by multiplying the work per ascent by the mean of the number of completed climbs.

Muscle power (*P*) was calculated as:

$$P\, = \,W/t.$$

where *t* corresponds to the mean time required to complete one ascent (s). Power values were expressed in watts (*W*) and converted to milliwatts (mW).

The values obtained for work (*W*) and power (*P*) were normalized to the body mass of each animal (g) to account for differences in body size between males and females.

**Table 1:** Mean functional performance and muscle mechanics during ladder-climbing exercise in male and female mdx mice across training days

Variable	Male	Female	Unit
Animal weight	22.0	20.3	g
External load	0.00874	0.00874	kg
Vertical displacement per ascent	0.689	0.689	m
Work per ascent	0.059	0.059	J
Mean set time	524.9 ± 86.9	724.4 ± 85.9	s
Mean ascent time	79.6 ± 6.7	75.0 ± 7.0	s
Mean power per ascent	0.043 ± 0.008	0.088 ± 0.001	mW
Mean number of climbs/session	6.3 ± 0.8	9.4 ± 0.6	climbs
Total work/session	0.017 ± 0.002	0.027 ± 0.001	J

g, kilogram; m, meter; J, joule; s, second; mW, milliwatt;

Example calculations using the mean values presented in Table 1: 

Male:

$${\mathrm{W}}\, = \,0.00{\mathrm{874}}\, \times \,{\mathrm{9}}.{\mathrm{81}}\, \times \,0.{\mathrm{689}}.$$

$${\mathrm{W}}\, = \,0.0{\mathrm{59}}~{\mathrm{J}}.$$

$${\text{total W}}\, = \,0.0{\mathrm{59}}~{\text{J x 6}}.{\text{3 }}\left( {{\text{mean number of climbs}}/{\mathrm{session}}} \right).$$

$${\text{total W}}\, = \,0,{\mathrm{371}}.$$

$${\text{total W}}/{\mathrm{g}}\, = \,0,{\mathrm{371}}/{\mathrm{22}}~{\text{g }}\left( {{\text{mean of animal weight}}} \right).$$

$${\text{total W}}/{\mathrm{g}}\, = \,0.0{\mathrm{17}}~{\mathrm{J}}/{\mathrm{g}}.$$

$${\mathrm{P}}\, = \,{\mathrm{W}}/{\mathrm{t}}.$$

$$P\, = \,0.0{\mathrm{59}}~{\mathrm{J}}/{\mathrm{79}}.{\mathrm{6}}~{\mathrm{s}}.$$

$$P\, = \,0.000{\mathrm{9}}~{\mathrm{W}}.$$

$${\mathrm{P}}/{\mathrm{g}}\, = \,0.000{\mathrm{9}}/{\mathrm{22}}~{\mathrm{g}}.$$

$${\mathrm{P}}/{\mathrm{g}}\, = \,0.0000{\mathrm{4}}~{\mathrm{W}}.$$

$${\mathrm{P}}/{\mathrm{g}}\, = \,0.0{\text{4 mW}}.$$

Female:

$${\mathrm{W}}\, = \,0.00{\mathrm{874}}\, \times \,{\mathrm{9}}.{\mathrm{81}}\, \times \,0.{\mathrm{689}}$$

$${\mathrm{W}}\, = \,0.0{\mathrm{59}}~{\mathrm{J}}.$$

$${\text{total W}}\, = \,0.0{\mathrm{59}}~{\text{J x 9}}.{\text{4 }}\left( {{\text{mean number of climbs}}/{\mathrm{session}}} \right).$$

$${\text{total W}}\, = \,0,{\mathrm{554}}.$$

$${\text{total W}}/{\mathrm{g}}\, = \,0,{\text{ 554}}/{\mathrm{2}}0.{\mathrm{3}}~{\text{g }}\left( {{\text{mean of animal weight}}} \right).$$

$${\text{total W}}/{\text{g W}}\, = \,0.0{\mathrm{27}}~{\mathrm{J}}.$$

$${\mathrm{P}}\, = \,{\mathrm{W}}/{\mathrm{t}}.$$

$$P\, = \,0.0{\mathrm{59}}~{\mathrm{J}}/{\mathrm{75}}.0~{\mathrm{s}}.$$

$$P\, = \,0.000{\mathrm{8}}~{\mathrm{W}}.$$

$${\mathrm{P}}/{\mathrm{g}}\, = \,0.000{\mathrm{8}}/{\mathrm{2}}0.{\mathrm{3}}~{\mathrm{g}}.$$

$${\mathrm{P}}/{\mathrm{g}}\, = \,0.0000{\mathrm{4}}~{\mathrm{W}}.$$

$${\mathrm{P}}/{\mathrm{g}}\, = \,0.0{\text{4 mW}}.$$

### Serum creatine kinase (CK)

Twenty-four hours after the function test, the animals in each experimental group were anesthetized intraperitoneally with a solution of 10 mg/kg body weight of 2% xylazine hydrochloride (Vyrbaxyl, Virbac) and 100 mg/kg body weight of ketamine hydrochloride (Francotar, Virbac). Subsequently, blood samples were collected by cardiac puncture and used to determine CK activity. The function test had been performed 24 h after the last exercise session, resulting in blood collection approximately 48 h after the final exercise bout.

The samples were centrifuged (DAIKI^®^ DTC16000 centrifuge) at 3000 rpm for 10 min at 4 °C. The serum obtained was used to determine CK activity using the Bioclin CK Nac Kinetic Crystal kit. The absorbances of the samples were read at 25 °C using a Genesys 10-S spectrophotometer (Thermo Electronic Corporation) with a wavelength of 340 nm.

### Histopathological Analysis

After anesthetic administration and blood withdrawal, the animals were perfused with PBS to minimize the influence of plasma components on subsequent analyses. Once death was confirmed, the biceps brachii (BB) and diaphragm (DIA) muscles were removed, initially frozen in isopentane at -90 °C, previously cooled in liquid nitrogen, and subsequently immersed in liquid nitrogen at -159 °C, being stored in a biofreezer at -70 °C. The DIA is one of the most severely affected muscles in the mdx model and is commonly used to assess disease progression in mdx mice (Grounds et al. 2008). The BB was included due to its functional recruitment during forelimb-dependent exercise such as ladder-climbing.

To obtain sections using a cryostat (Leica Biosystems CM1520), the muscles were kept at -23 °C, sectioned transversely at a thickness of 8 μm, and collected on slides. Slides containing 12 sections from each experimental group were prepared for staining with Hematoxylin and Eosin (HE) and Picrosirius Red (PS).

The HE-stained slides were observed under a light microscope, and the number of muscle fibers with a central nucleus (indicative of regeneration), the number of fibers with a peripheral nucleus (characteristic of normal fibers), the areas of inflammatory infiltrate, and the total area of the muscle were evaluated (Grounds 2010).

Slides stained with Picrosirius Red were analyzed under a light microscope using polarized light, with the aim of identifying and quantifying the area of fibrosis in the muscle tissue. The sections were immersed in Picrosirius Red dye, washed in running water to remove excess, counterstained with hematoxylin, washed again in running water, dehydrated in increasing degrees of ethyl alcohol, immersed in xylene, and mounted on slides.

The images of the histological sections (HE and PS) were captured using a computerized image analysis system (AxioVision V4.8.2.0 software, White Plains, NY, USA) coupled to the ZEISS Scope A1^®^ microscope, and the morphometric analyses were performed using ImageJ 1.54 g (Bethesda, MD, USA) and Image-Pro Plus V6.3^®^ (Rockville, MD, USA) software.

### Statistical Analysis

All data were expressed as mean ± standard error of the mean (SE). Data distribution was assessed using the Shapiro–Wilk test prior to statistical comparisons. Differences between groups were analyzed using two-way ANOVA to evaluate the effects of sex and exercise modality, followed by Tukey’s multiple comparisons test. Statistical analyses were performed using GraphPad Prism (GraphPad Software Inc., San Diego, CA, USA). P-values ≤ 0.05 were considered statistically significant.

## Results

### Sex differences in muscle damage in mdx mice

**Fig. 2:**
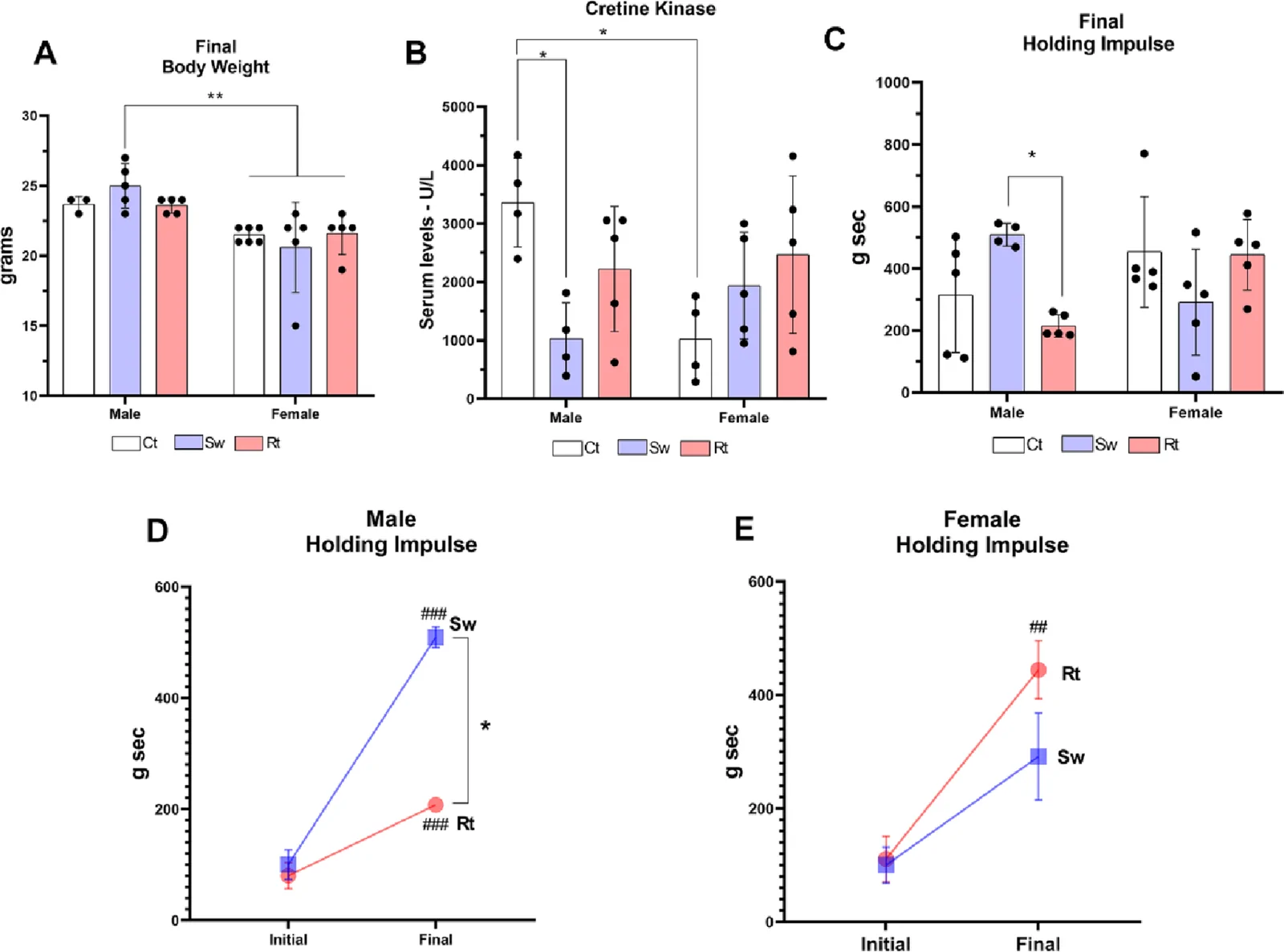
Muscle function and damage assessment in mice subjected to the different experimental protocols. (**A**) Final body weight of male and female animals in the control (Ct), swimming (Sw), and resistance training (Rt) groups. (**B**) Serum creatine kinase levels (U/L) in the same groups, indicating the response to physical effort. (**C**) Final holding impulse (g·sec) in males and females. (**D**) Evolution of holding impulse between initial and final time points for males. (**E**) Evolution of holding impulse between initial and final time points for females. Significance symbols indicate statistically relevant differences between groups (* < 0.05; ** < 0.001) and/or time points (## < 0.001; ### < 0.0001). (Two-way ANOVA with Tukey’s post hoc test). Main effects of sex, exercise, and their interaction (sex × exercise) were analyzed

Greater muscle damage in mdx males, compared to females, was evidenced by significantly higher serum CK levels observed in the MCt group (3360.4 ± 760.1 U/L) compared to the FCt group (1024.0 ± 702.2 U/L, *p* = 0.026, Fig. 2B), representing an increase of approximately 228%.

At the end of the exercise protocol, male mdx mice in the MSw group (23.6 ± 0.5 g) showed higher body weight compared to female groups, consistent with the expected sexual dimorphism in mice (FCt: 21.5 ± 0.5 g, *p* = 0.022; FSw: 20.6 ± 3.2 g, *p* = 0.004; FRt: 21.8 ± 1.4 g, *p* = 0.038; Fig. 2A). No significant differences were observed among male experimental groups at the end of the protocol. 

### Effects of exercise modality on muscle damage and function

In parallel, males subjected to swimming exercise (MSw group) showed a significant reduction in CK levels (1027.8 ± 615.0 U/L, *p* = 0.026) compared to the MCt group (reduced by approximately 69%; Fig. 2B).

No significant differences in holding impulse were observed between exercised and sedentary male groups at the end of the protocol. However, swimming-trained males exhibited significantly greater holding impulse (509.3 ± 36.6 g sec), approximately 137% higher, compared to the MRt group (215.0 ± 36.3 g sec, *p* = 0.043; Fig. 2D). Considering the evolution of holding impulse throughout the experimental period, the MSw (gain of approximately 400%, *p* < 0.0001) and FRt (gain of approximately 300%, *p* = 0.001) groups showed a significant gain in holding impulse relative to their respective baseline values throughout the experimental period (Fig. 2D-E).

### Interaction between sex and exercise on muscle histopathology

**Fig. 3:**
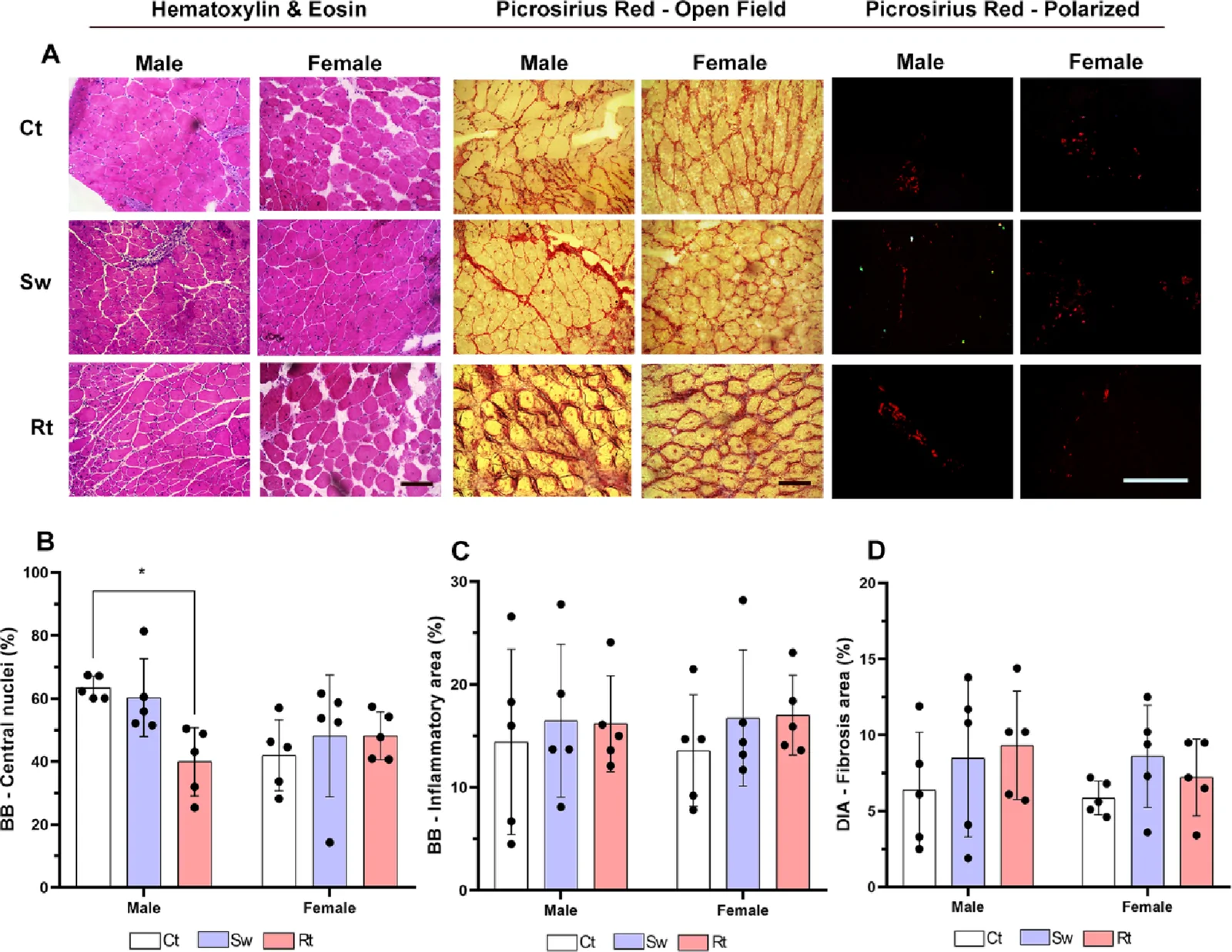
Histological assessment of biceps brachii muscle in mice subjected to the different experimental protocols. (**A**) Representative cross-sections of the biceps brachii muscle stained with Hematoxylin & Eosin (H&E) and Picrosirius Red (visualized under both brightfield and polarized light) from male and female animals in the Control (Ct), swimming (Sw), and resistance training (Rt) groups. Scale bar = 100 μm. (**B**) Quantification (expressed as a percentage) of central nuclei fibers; (**C**) inflammatory area; and (**D**) fibrotic area in the different experimental groups. Significance symbols indicate statistically relevant differences between groups (* < 0.05). (Two-way ANOVA with Tukey’s post hoc test). Main effects of sex, exercise, and their interaction (sex × exercise) were analyzed

**Fig. 4:**
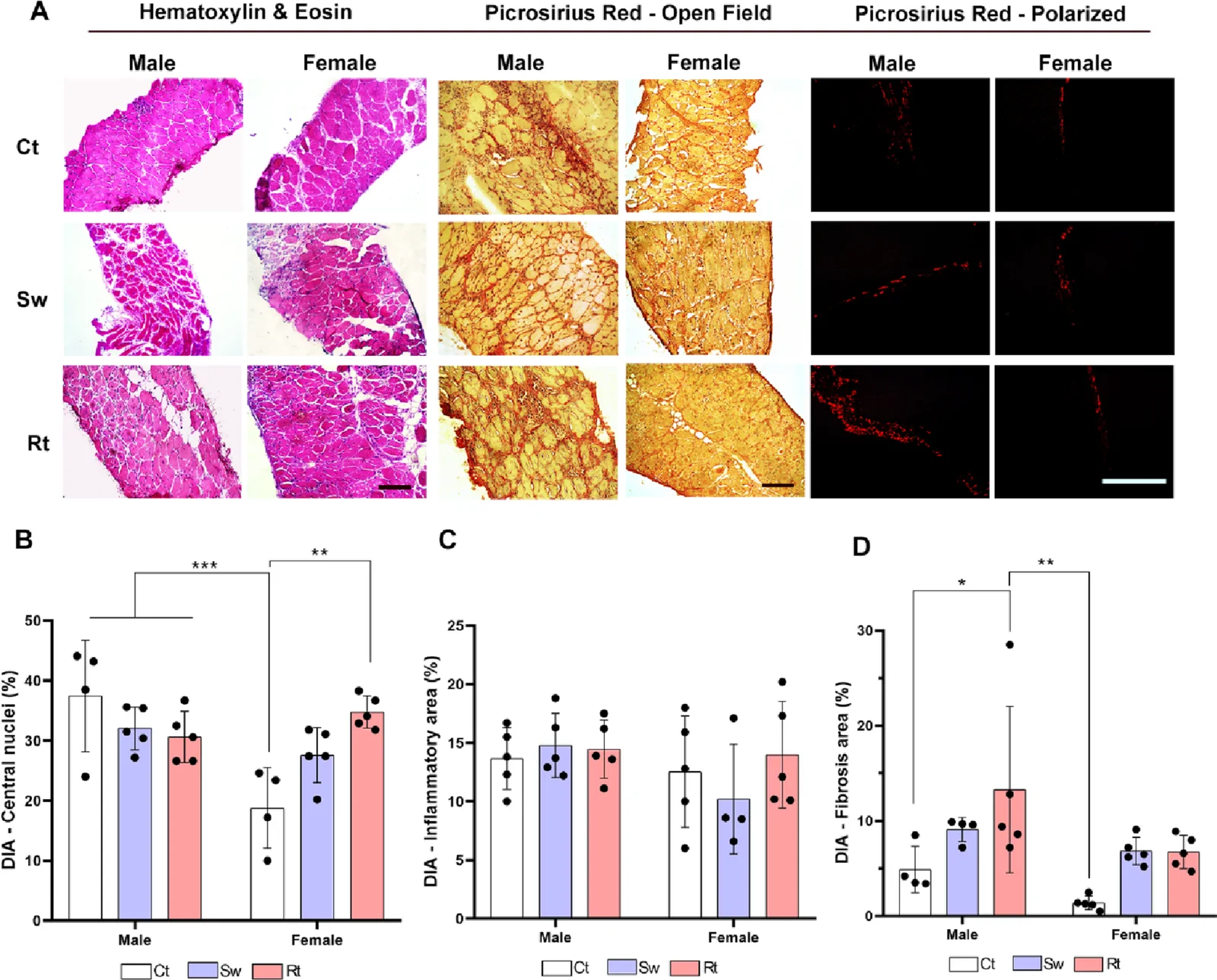
Histological assessment of diaphragm muscle in mice subjected to the different experimental protocols. (**A**) Representative cross-sections of the diaphragm muscle stained with Hematoxylin & Eosin (H&E) and Picrosirius Red (visualized under both brightfield and polarized light) from male and female animals in the Control (Ct), swimming (Sw), and resistance training (Rt) groups. Scale bar = 100 μm. (**B**) Quantification (expressed as a percentage) of central nuclei fibers; (**C**) inflammatory area; and (**D**) fibrotic area in the different experimental groups. Significance symbols indicate statistically relevant differences between groups (* < 0.05). (Two-way ANOVA with Tukey’s post hoc test). Main effects of sex, exercise, and their interaction (sex × exercise) were analyzed

Histopathological analysis showed that, in the BB muscle, resistance exercise on a ladder significantly reduced the number of muscle fibers with a central nucleus in male mdx (MRt: 39.9 ± 10.8%), representing a 37.1% reduction compared to the MCt group (63.4 ± 3.6%, *p* = 0.045; Fig. 3B). Female sedentary mdx mice (FCt 18.8 ± 6.7%) exhibited lower percentages of centrally nucleated fibers in the DIA compared to male groups (an approximate 38–50%; MCt: 37.4 ± 9.3%, *p* = 0.0008; MSw: 32.0 ± 3.5%, *p* = 0.014; MRt: 30.5 ± 4.2%, *p* = 0.034; Fig. 4B). After the application of the ladder-climbing exercise, female mdx (FRt: 34.7 ± 2.7%) showed a significant increase in the population of fibers with a central nucleus compared to the FCt group, representing a 85.6% increase (*p* = 0.002; Fig. 4B). Additionally, in male mdx mice, DIA fibrosis was higher in the MRt group (6.7 ± 1.7%) compared to sedentary controls (MCt: 4.9 ± 2.4%, *p* = 0.047; FCt: 1.5 ± 0.6%, *p* = 0.001; Fig. 4D). 

### Sex differences in performance during resistance exercise

**Fig. 5:**
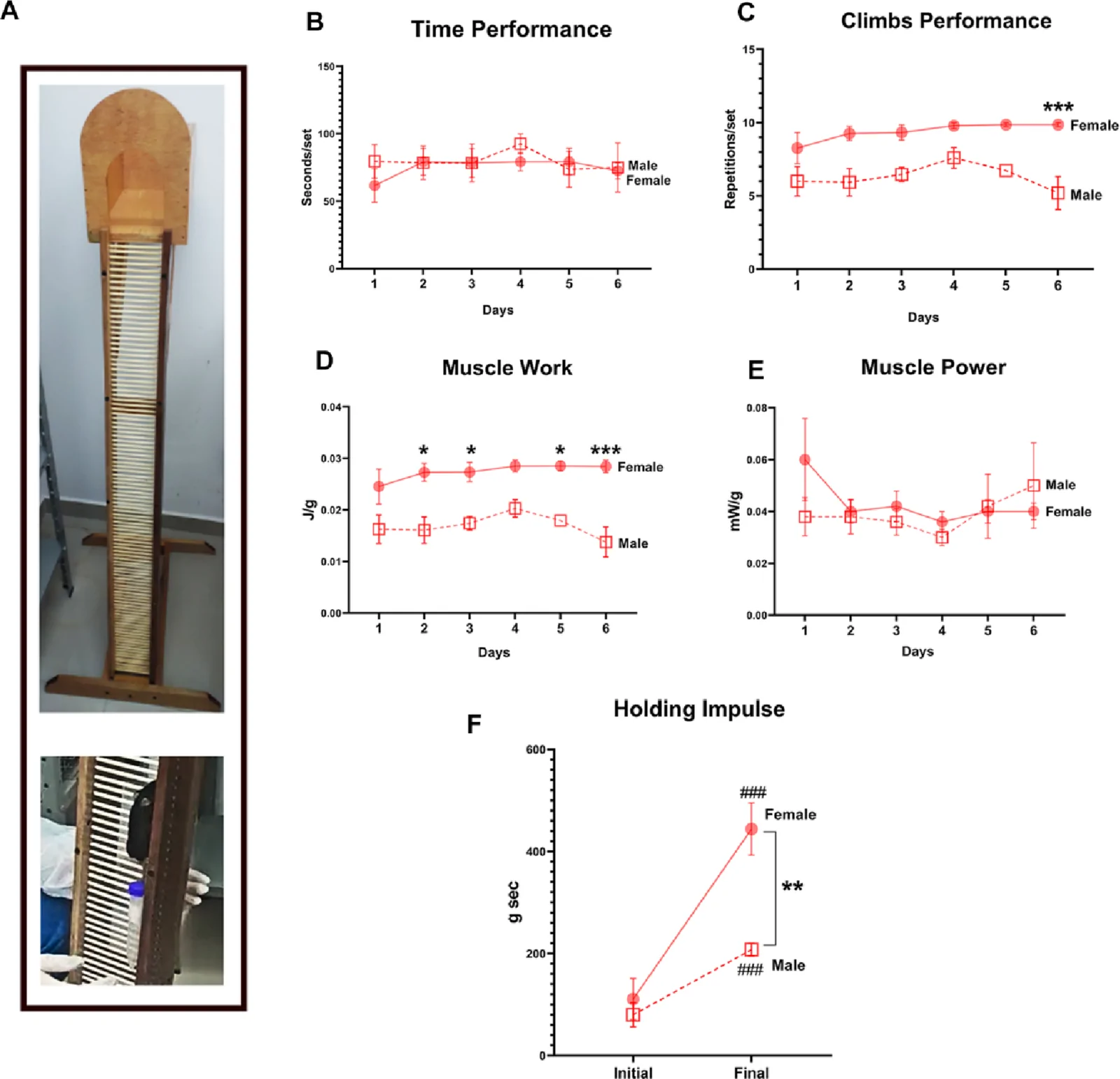
Temporal analysis of performance and functional parameters in mice subjected to resistance training protocol (Rt). (A) Inclined ladder (80°, 100 cm long, 9 cm wide) containing metal bars spaced every 0.5 cm. (**B**) Time Performance: Evolution of task completion time (in seconds) over the experimental period for male and female animals. (**C**) Climbs Performance: Number of successful climbs performed over the experimental days for male and female animals. (**D**) Muscle Work: Calculated muscle work (J/g) for male and female animals at the final assessment point. (**E**) Muscle Power: Peak muscle power (mW/g) generated by male and female animals at the final assessment point. (**F**) Holding Impulse: Impulse (in g sec) sustained during the holding task for male and female animals at the final assessment point. Significance symbols indicate statistically relevant differences between groups (* < 0.05; ** < 0.001; *** < 0.0001) and/or time points (## < 0.001; ### < 0.0001). (Two-way ANOVA with Tukey’s post hoc test). Main effects of sex, exercise, and their interaction (sex × exercise) were analyzed

Performance analyses were restricted to resistance-trained groups because sedentary and swimming animals were not exposed to the ladder-climbing protocol. Under these conditions, significant differences in performance were observed between males and females across the six training sessions (Fig. 5). The average time spent per climb did not differ significantly between males and females over the six training days (Fig. 5B). Both groups maintained relatively stable values, indicating that the speed of task execution was similar between the sexes. Females consistently maintained the number of repetitions per set, while males exhibited inferior performance throughout the protocol (Fig. 5C). The difference became statistically significant on the last day, when females performed a significantly higher number of climbs compared to males (MRt: 5.2 ± 1.6 vs. FRt: 8.5 ± 1.6 climbs/set, 63% more climbs/set, *p* = 0.0008; Fig. 5C). This result indicates greater tolerance to repetitive effort and greater fatigable resistance in the female group.

Mechanical work was greater in females across virtually all sessions analyzed. Significant differences were observed on days 2 and 3 and remained evident on day 6 (MRt: 0.014 ± 0.004 vs. FRt: 0.024 ± 0.004 J/g, 71.4% greater, *p* = 0.0003; Fig. 5D). This pattern indicates that females produced more mechanical work per session, a direct reflection of the greater number of repetitions completed without a proportional increase in the average time per climb.

Despite differences in total mechanical work, no significant differences were observed between the sexes regarding normalized average power (Fig. 5E). The values fluctuated similarly between males and females, suggesting that both produced work at the same average rate. This finding reinforces that the better performance of females did not result from greater muscle power, but rather from a greater ability to sustain the same power for longer, resulting in superior accumulated work.

Finally, the holding impulse increased significantly from the beginning to the end of the experiment in both sexes, suggesting progressive improvement in task performance throughout the protocol (Fig. 5F). However, females had 106.6% higher final values than males (MRt: 215.0 ± 36.3 g sec vs. FRt: 444.2 ± 114.4 g sec, *p* = 0.001; Fig. 5F).

This result indicate that females can withstand the load for a substantially longer time, supporting the observation that females sustained the task for longer periods under the present experimental conditions.

## Discussion

In the present study, swimming reduced serum CK levels in male mdx mice compared to controls and also improved their holding impulse relative to males subjected to the loaded ladder-climbing protocol. Swimming is often considered a moderate/low-intensity modality capable of inducing beneficial effects, such as reduced inflammatory infiltrate and the promotion of muscle regeneration (Frinchi et al. 2021). The observed effects of swimming may be attributed to adaptive responses induced under low-demand conditions. Training sessions of 30/day for 4 consecutive days induced an increase in serum CK levels and increased myonecrosis in mdx mice (Matsakas et al. 2013). In the study of Hall et al. (2007) male mice subjected to a protocol consisting of four weeks of pre-training with a progressive increase from 8 to 12 m/min, followed by six weeks of moderate-intensity training (12 m/min, 30 min/day), showed a reduction in serum CK levels, along with muscle adaptations in the extensor digitorum longus, gastrocnemius, and quadriceps muscles. Thus, the present findings suggest that dystrophic muscle adaptations may depend strongly on exercise intensity, duration, and progression rather than exclusively on exercise modality. The present study should be interpreted as an exploratory investigation of sex-dependent exercise adaptations in mdx mice.

Although limited by sample size, the study identified consistent functional and histopathological trends across distinct exercise modalities. Isometric resistance paradigms and mechanical loading approaches have previously been investigated in mdx mice (Lindsay et al. 2019; Yamauchi et al. 2023, 2025), demonstrating functional and structural adaptations. Our results demonstrate that male mice trained with loaded ladder-climbing exhibit increased fibrosis in the DIA. However, this increase was tissue-specific and did not result in a decrease in overall muscle function test or a significant elevation in systemic CK levels compared to sedentary controls. These results align with those of Hyzewicz et al. (2015), which demonstrated that high-intensity protocols can increase fibrosis, particularly in muscles with high functional demands, such as in the DIA, which operates in a state of continuous overload and exhibits severe inflammation and fibrosis as hallmark characteristics of the dystrophy (Hyzewicz et al. 2015a; Kharraz et al. 2014; Radley and Grounds 2006). Furthermore, although mdx mice naturally exhibit greater regenerative capacity, contributing to a milder form of dystrophy, this phenomenon is not observed in DIA, whose regeneration after muscle necrosis does not restore its original structure (Louboutin et al. 1993). According to Duddy et al. (2015), in mdx mice, myogenic mechanisms are sufficiently competent to maintain muscle mass even in the presence of massive and persistent myofiber degeneration. In contrast, human dystrophic muscle fibers lose much of their regenerative capacity due to satellite-cell depletion (Sacco et al. 2010). In this context, the reduction in the number of core fibers in the BB of resistance-trained male animals, without worsening of functional parameters, may suggest a stabilization of the degeneration-regeneration cycle. Taken together, these findings indicate that the climbing protocol appears to represent a feasible and safe model for inducing controlled mechanical stress, making it potentially useful for future studies investigating regenerative, pharmacological, or genetic therapies in the mdx model. A limitation of the present study is the relatively small sample size, which reflects the inherent variability of in vivo mdx studies and should be considered when interpreting the results. In addition, the absence of a healthy control group should be considered when interpreting the findings, as the study was specifically designed to compare different exercise modalities within the mdx mouse. Another limitation of this study is that exercise intensity was not individualized based on each animal’s maximal capacity, which should be considered when interpreting the results. Thus, future studies may consider increasing the sample size, the number of training sets, and/or the applied load to produce muscle damage in the mdx model that more closely resembles that observed in human disease.

The results of this study highlight sex as a critical factor in modulating the pathophysiology of dystrophy in mdx models in response to physical exercise, a finding that corroborates our previous studies (Hermes et al. 2018). As observed, females subjected to both exercise protocols exhibited a consistently less severe phenotype compared to males. The literature attributes this advantage, in large part, to the protective role of estrogen (Nascimento et al. 2025; Timpani et al. 2024). This hormone is widely recognized for its ability to modulate the inflammatory response, protect muscle membrane integrity against injury (especially calcium influx-mediated injury), and reduce oxidative stress (Zorov et al. 2014), providing a more favorable physiological condition. The lack of functional dystrophin weakens the sarcolemma, leading to successive episodes of myonecrosis, oxidative stress, inflammation, and muscle regeneration, culminating in the progressive accumulation of fibrosis (Grounds et al. 2020). This study suggests that physiological hormonal factors may contribute to the reduced fibrotic response observed in exercised females undergoing resistance exercise. These findings are consistent with previous evidence suggesting a protective role of estrogen in dystrophic muscle. The maintenance of serum CK levels and the significant gain in holding impulse observed in the FRt group further support the interpretation that resistance training was well-tolerated and promoted positive functional adaptations in mdx females.

Evaluation of mdx mice during the loaded ladder-climbing protocol also indicate that female mice were more resistant to fatigue (Ferraresi et al. 2015). Compared to male mdx mice, females exhibited greater total mechanical work at constant average power, indicating that they were able to sustain exercise for a longer period. These findings agree with previous studies in mdx mice, in which females demonstrated better preservation of tetanic strength compared to males (Hakim and Duan 2012) and that estrogen deficiency in mdx females impairs post-exercise strength recovery (Vang et al. 2021). Differences in fatigue resistance between males and females are known to be task-dependent, varying according to the type of task, muscle group, and exercise modality. In several conditions, women/females demonstrate greater fatigable resistance, especially in submaximal and sustained tasks (Avin and Law 2011; Temesi et al. 2015), which reinforces the idea that mdx females may naturally have greater functional endurance or favorable adaptation to exercise. It is important to consider that DMD is an X-linked disorder that predominantly affects males (Duan et al. 2021). Although both male and female mdx mouse were included in the present study to explore potential sex-related differences in muscle adaptation to exercise, caution is required when translating these findings directly to the clinical context.

Thus, given the increased susceptibility to fatigue and the marked lifelong muscle weakness characteristic of mdx mice (Grounds et al. 2008; Lynch et al. 2001), the application of tests capable of challenging the musculature through repeated stimuli is sensitive in revealing deficits in contractile function and resistance to fatigue. Several studies using different functional protocols converge on this same pattern of vulnerability. Fatigue protocols with repeated tetany (Call et al. 2008), 10-minute in situ fatigue tests of the anterior tibial muscle (Tamayo et al. 2016), assessments of contraction and torque after acute and chronic exercise (Capogrosso et al. 2016) and treadmill exhaustion tests (Hamm et al. 2021) confirm the sensitivity of repetitive performance-based measures not only for detecting functional decline, but also for demonstrating functional improvements following exercise or therapeutic interventions. Taken together, these findings support the idea that functional methods integrating strength, fatigue, and work capacity provide complementary functional insights into the muscular phenotype of the mdx. Therefore, the findings should be interpreted cautiously and primarily as evidence of biological trends and feasibility rather than definitive effect estimates.

In conclusion, light to moderate/low-intensity exercise (swimming) appeared to promote protective and functional adaptations, reducing systemic damage markers (CK) in males. In contrast, resistance exercise (ladder-climbing) induced measurable muscle adaptations, including localized fibrotic responses in the male DIA, while it promoted significant functional gains in female mdx mice. These findings suggest the importance of defining the threshold between adaptive stimulation and excessive mechanical loading in DMD. Furthermore, beyond structured exercise paradigms, voluntary physical activity (such as wheel running or spontaneous climbing behavior) may represent an additional and potentially beneficial dimension of physical training in the mdx model, warranting further investigation. The loaded ladder-climbing protocol may represent a feasible and complementary functional approach for investigating exercise responses in the mdx model.

## Data Availability

All data generated and included in this study can be requested by contacting the corresponding author.

## Abbreviations

DMD: Duchenne Muscular Dystrophy
mdx: Modelo murino distrófico (C57BL/10-Dmdmdx)
BB: Biceps brachii
DIA: Diafragma
CK: Creatine kinase
HE: Hematoxilina e Eosina
PS: Picrosirius Red
MCt / FCt: Male/Female Control (Sedentary)
MSw / FSw: Male/Female Swimming
MRt / FRt: Male/Female Resistance Training
W: Mechanical Work
P: Power
